# Elevated HMGB1 Levels in Neonates With Bronchopulmonary Dysplasia: A Systematic Review and Meta‐Analysis

**DOI:** 10.1155/carj/2816142

**Published:** 2026-08-13

**Authors:** Hejia Li, Jiao Li, Shanshan Li, Xiaojuan Huang, Shuaiyi Li, Wenhui Wu, Yuqing Fan, Yurun Wang, Yue Zhang, Jiashun Liu, Lu Zou, Changjun Ren, Shangbin Li

**Affiliations:** ^1^ Department of Pediatrics, First Hospital of Hebei Medical University, Shijiazhuang 050000, China, hebmu.edu.cn; ^2^ Department of Pediatrics, Bayingolin Mongolian Autonomous Prefecture People’s Hospital, Korla 841000, China; ^3^ Department of Rodent Biology, Chifeng City Center for Disease Control and Prevention, Chifeng 024000, China

**Keywords:** bronchopulmonary dysplasia, diagnosis, high-mobility group box 1, meta-analysis, neonates

## Abstract

**Background:**

To assess the clinical relevance of high‐mobility group box 1 (HMGB1) in bronchopulmonary dysplasia (BPD), we conducted a systematic review and meta‐analysis comparing HMGB1 levels in neonates with and without BPD.

**Methods:**

Eight databases (PubMed, Cochrane Library, Web of Science, Embase, CNKI, VIP, Wanfang, and CBM) were searched up to March 1, 2025. Cohort studies reporting serum or bronchoalveolar lavage fluid (BALF) HMGB1 levels in neonates were included. Meta‐analysis was performed using RevMan 5.4.

**Results:**

Seven studies (*n* = 527 neonates; 308 BPD, 219 controls) were included. HMGB1 concentrations in BALF were significantly higher in neonates with BPD than in those without BPD (mean difference [MD], 5.04 ng/mL; 95% confidence interval [CI], 4.09–5.99; *p* < 0.00001). Serum HMGB1 concentrations were also higher in neonates with BPD (MD, 7.24 ng/mL; 95% CI, 4.53–9.96; *p* < 0.00001). Subgroup analyses across postnatal time points indicated that serum HMGB1 levels tended to be higher in infants with BPD than in controls; however, the progressive increase observed from Stage I to Stage III plateaued thereafter, with no further increase in Stage IV (> 20 days).

**Conclusion:**

Total HMGB1 concentrations in serum and BALF are elevated in neonates with BPD, which may reflect BPD‐associated inflammatory lung injury. However, the clinical utility of HMGB1 is limited by its lack of disease specificity and the inability of current assays to distinguish among biologically distinct redox isoforms. Further prospective studies are needed to clarify its diagnostic and therapeutic relevance.

## 1. Introduction

Bronchopulmonary dysplasia (BPD) is the most common chronic lung disease in premature infants with low birth weight, often affecting those who require postnatal respiratory support [1, 2]. BPD is strongly associated with long‐term respiratory complications and neurodevelopmental impairment, and it carries a significant healthcare burden [3]. Although the survival rate of preterm infants has improved markedly with advances in neonatal intensive care, the incidence of BPD has remained unchanged or is even increasing [4].

However, the pathogenesis of BPD remains incompletely understood, and it involves a constellation of biological processes, including inflammation, oxidative stress, impaired alveolarization, and microvascular dysplasia [5]. Recent studies have suggested a significant role of sterile inflammation in lung injury in BPD, potentially driven by activation of the innate immune response through endogenous damage‐associated molecular pattern proteins [6]. High‐mobility group box 1 (HMGB1), a highly conserved nuclear protein, is released extracellularly in response to cellular injury or stress and functions as a prototypical damage‐associated molecular pattern [7]. Importantly, extracellular HMGB1 acts as a potent pro‐inflammatory mediator that amplifies innate immune activation, largely through its engagement of pattern‐recognition receptors. Its immunological activity is tightly regulated by biochemical states: The all‐thiol form promotes chemotaxis, the disulfide form drives cytokine release, and terminal oxidation renders it inactive [8]. Post‐translational modifications (e.g., acetylation and phosphorylation) facilitate HMGB1 nuclear export and secretion [9]. Extracellular HMGB1 engages pattern‐recognition receptors, including Toll‐like receptor‐2 (TLR2), TLR4, TLR9, and receptor for advanced glycation end‐products (RAGE), thereby activating downstream myeloid differentiation primary response 88/TIR‐domain‐containing adapter‐inducing interferon‐β pathways and nuclear factor kappa‐light‐chain‐enhancer of activated B cells (NF‐κB; [10–12]) and amplifying inflammatory signaling relevant to BPD pathogenesis [13]. Emerging evidence has implicated HMGB1 in the pathophysiology of neonatal pulmonary diseases [14, 15]. In preclinical models of BPD, systemic administration of HMGB1‐targeting peptides markedly attenuated lung injury. These peptides exert anti‐inflammatory and antifibrotic effects by suppressing pro‐inflammatory cytokine release and reducing soluble collagen deposition in lung tissue [16]. Such findings suggest a potential therapeutic role for HMGB1 modulation in preventing or ameliorating BPD‐associated lung injury.

Clinical studies have further supported a link between elevated HMGB1 levels and BPD in neonates, suggesting the utility of HMGB1 as an early biomarker [17]. BPD follows four postnatal stages, namely, Stages I–IV, each reflecting evolving inflammatory, epithelial, and vascular pathology. Because HMGB1 participates in early sterile inflammation and potentially exhibits stage‐specific kinetics, defining its temporal profile is essential. However, current diagnostic evidence is constrained by small sample sizes, methodological heterogeneity, and limited mechanistic understanding. Moreover, published studies have reported inconsistent findings regarding the magnitude and timing of HMGB1 elevation, and the clinical relevance of these differences, particularly their diagnostic or prognostic value, remains uncertain.

To address these gaps, we conducted a systematic review and meta‐analysis to compare total HMGB1 concentrations between neonates with and without BPD, characterize their temporal patterns, and evaluate the potential clinical relevance and limitations of HMGB1 as a marker of BPD‐associated inflammatory lung injury.

## 2. Materials and Methods

### 2.1. Literature Search

A comprehensive search was conducted across eight electronic databases—PubMed, Cochrane Library, Web of Science, Embase, CNKI, VIP, Wanfang, and the Chinese Biomedical Literature Database—to identify studies assessing HMGB1 levels in serum and bronchoalveolar lavage fluid (BALF) in neonates with BPD. The search covered all records from database inception to March 1, 2025. Search terms were constructed using Boolean operators as follows: (HMGB1 OR “high mobility group box‐1 protein”) AND (“bronchopulmonary dysplasia” OR BPD OR “chronic lung disease of prematurity”) AND (“preterm infant” OR “premature infant”). In addition, the reference lists of eligible articles published within the past 5 years, as well as recent publications from *Pediatric Research* and *Journal of Perinatology*, were manually screened to identify additional relevant studies. The study protocol has been registered in the PROSPERO database (CRD42025634267).

### 2.2. Study Selection

Literature management was performed using NoteExpress, with duplicates removed through an automated process. Two reviewers independently screened the titles and abstracts of all retrieved records, followed by a full‐text evaluation of potentially eligible studies. Studies were included if they met the following criteria: preterm infants born at < 37 weeks’ gestation; BPD diagnosed using international consensus, the 2018 National Institute of Child Health and Human Development definition, or Jobe–Bancalari severity criteria [18]; HMGB1 levels measured in serum/plasma or BALF with a specified quantitative method; results reported as the mean ± standard deviation (SD) or median with interquartile range; and prospective or retrospective design. Meanwhile, the following studies were excluded: case reports, conference abstracts, reviews, or other sources lacking primary data; studies involving infants with major congenital anomalies or metabolic disorders; studies lacking standardized BPD diagnostic criteria; duplicate reports or studies containing incomplete data; and studies not published in English or Chinese. The literature search strategy was independently executed by two researchers (Hejia Li and Shanshan Li). Disagreements during the retrieval process were resolved through negotiation. Any persisting disagreements were resolved through arbitration by a third researcher (Shangbin Li).

### 2.3. Data Collection

Data were extracted for the following variables: first author, publication year, and country; study population characteristics, including sample size, gestational age, and birth weight; type of specimen used for HMGB1 measurement; and effect size data (mean and SD). For data reported as medians with interquartile ranges, means and SDs were estimated using Wan’s method [19], and subgroup data were combined as appropriate. When necessary, data points presented in figures were extracted using Engauge Digitizer.

### 2.4. Quality Assessment

The methodological quality of included studies was independently assessed by two authors using the Newcastle–Ottawa Scale, which evaluates three domains: selection of study population (four items), comparability between groups (two items), and outcome assessment (three items). The maximum score is 9, with scores of ≤ 4, 5–6, and ≥ 7 denoting low, moderate, and high quality, respectively. Discrepancies were resolved through consultation with a third reviewer.

### 2.5. Statistical Analysis

Data analysis was performed using RevMan 5.4. For continuous outcomes, effect sizes were reported as mean differences (MDs) with 95% confidence intervals (CIs). When data were non‐normally distributed, standardized MDs were used. Heterogeneity was assessed using the *Q* test (*α* = 0.1) and *I* ^2^ statistic, with *I* ^2^ > 50% indicating substantial heterogeneity. A fixed‐effects model (Mantel–Haenszel) was applied when *I* ^2^ ≤ 50% and *p* ≥ 0.10; otherwise, a random‐effects model (DerSimonian–Laird) was used. Subgroup and sensitivity analyses were conducted to explore potential sources of heterogeneity. Statistical significance was defined as *p* < 0.05. Publication bias was assessed using funnel plots and Begg’s test.

## 3. Result

### 3.1. Literature Selection

**FIGURE 1:**
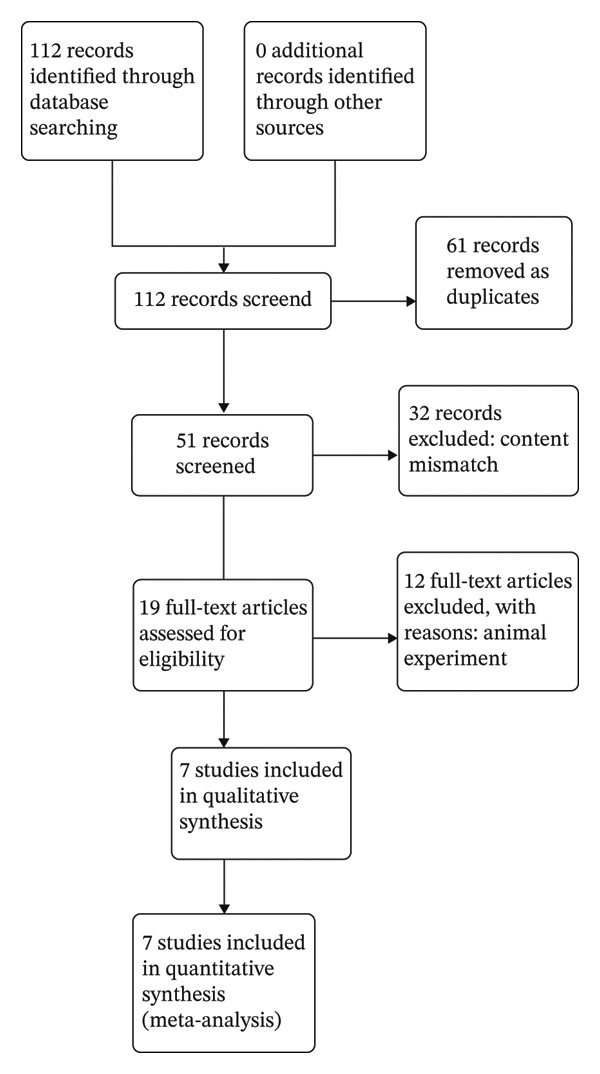
The flow diagram of study selection for the systematic review.

In total, 112 records were identified through database searches (Figure 1). After removing duplicates (*n* = 61), 51 unique records remained. These were screened by title and abstract, resulting in the exclusion of 32 records because of irrelevance to the study topic. The remaining 19 full‐text articles were assessed for eligibility, of which 12 were excluded, primarily because they were animal studies. Ultimately, seven studies meeting the inclusion criteria were included in the final data synthesis.

### 3.2. Basic Characteristics

The baseline characteristics and results of the quality assessment of the included studies are presented in Table 1. All seven studies were cohort studies, and they encompassed 527 neonates, including 308 patients diagnosed with BPD and 219 non‐BPD controls. Two studies assessed HMGB1 levels in both serum and BALF [20, 21], employing the classical pathological staging system proposed by Northway et al. [3] that delineates four distinct postnatal phases: Stage I (days 1–3), Stage II (days 4–10), Stage III (days 10–20), and Stage IV (> 20 days). Two additional studies measured HMGB1 exclusively in BALF [17, 22]. Aghai et al. [17] measured HMGB1 levels on postnatal days 1, 3, and 7, but only pooled effect sizes were reported without time‐specific data. The remaining three studies measured HMGB1 in serum alone [6, 23, 24]. Of these, Yoshio Shima et al. [6] reported HMGB1 concentrations as quartile ranges rather than means or medians.

**TABLE 1:** Baseline characteristics and quality assessment of the included studies (*n* = 7).

Number	First author	Country	Year	Neonates	Specimen	Specimen HMGB1 (ng/mL)						NOS score
						BPD group			Non‐BPD group			
						*n*	Serum	BALF	*n*	Serum	BALF	
No. 1	ZH Aghai	USA	2010	GA < 32 weeks, mechanical ventilation	BALF	60 (174)^✱^		21.9 ± 12.7	24 (61)^✱^		12.7 ± 6.6	8

No. 2	Liu Xiaoqiao	China	2014	GA < 33 weeks	Serum, BALF	18	I (15.72 ± 1.97)	I (7.35 ± 0.56)	24	10.84 ± 1.42	4.77 ± 0.47	6
						17	II (18.56 ± 2.12)	II (8.81 ± 0.84)				
						21	III (21.03 ± 2.34)	III (9.78 ± 0.97)				
						20	IV (25.12 ± 2.65)	IV (12.35 ± 1.24)				

No. 3	Cheng hongbin	China	2016	GA < 37 weeks, mechanical ventilation > 1 week	BALF	28		8.21 ± 1.02	35		4.29 ± 0.61	5

No. 4	Huang Xiaoqi	China	2016	GA < 33 weeks	Serum, BALF	12	I (14.89 ± 2.13)	I (7.42 ± 0.62)	16	11.14 ± 1.54	4.55 ± 0.52	6
						11	II (17.42 ± 2.33)	II (8.38 ± 0.77)				
						14	III (20.82 ± 2.41)	III (9.53 ± 0.92)				
						14	IV (24.46 ± 2.47)	IV (12.25 ± 1.14)				

No. 5	Zhou Yang	China	2020	GA = 28–32 weeks, BW < 1500 g	Serum	35	^∗∗^D3 (19.6 ± 4.2)		51	D3 (14.1 ± 2.7)		7
							D7 (25.1 ± 5.9)			D7 (17.5 ± 3.6)		
							D14 (28.2 ± 6.0)			D14 (16.2 ± 3.0)		

No. 6	Yoshio Shima	Japan	2021	GA < 33 weeks	Serum	12	D1: 20.6 (10.69–30.97)		13	D1: 21.8 (10.41–41.3)		7
							D14: 15.7 (11.4–23.1)			D14: 10.6 (6.3–21.0)		
							D28: 19.1 (12.2–23.2)			D28: 6.1 (4.2–10.3)		

No. 7	Du Weiwei	China	2022	GA = 28–32 weeks, BW < 1500 g,	Serum	40	D1 (18.74 ± 3.45)		56	D1 (17.8 5 ± 1.93)		8
							D7 (23.34 ± 1.83)			D7 (20.75 ± 2.69)		
							D14 (27.30 ± 2.50)			D14 (18.91 ± 1.53)		
							D28 (20.64 ± 2.99)			D28 (15.90 ± 2.56)		

*Note:* The data are presented as either the mean and standard deviation or the median and interquartile range. BPD, bronchopulmonary dysplasia; BALF, bronchoalveolar lavage fluid.

Abbreviations: BW, body weight; GA, gestational age; HMGB1, high‐mobility group box 1; NOS, Newcastle–Ottawa Scale.

^∗^Number of collected samples.

^∗∗^Days after birth.

### 3.3. HMGB1 in BALF

**FIGURE 2:**
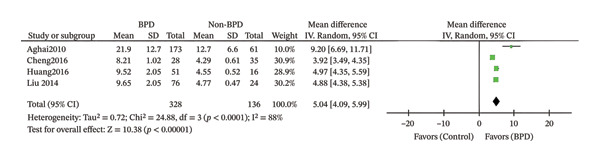
Forest plot comparing HMGB1 levels in BALF between the BPD and non‐BPD groups.

Four studies assessed HMGB1 levels in BALF. A significant degree of heterogeneity was observed among these studies (*I* ^2^ = 88%, *p* < 0.0001). Using a random‐effects model, the pooled MD was 5.04 ng/mL (95% CI = 4.09–5.99; *Z* = 10.38; *p* < 0.00001), indicating that HMGB1 levels were significantly higher in the BPD group than in the non‐BPD group (Figure 2). Sensitivity analysis, which was performed by sequentially excluding each study, demonstrated stable results with no material change in the pooled effect size (Supporting Table 1), supporting the robustness of the BALF findings. Subgroup analysis was not conducted because of the limited number of studies. The funnel plot appeared symmetric, and Begg’s test (*p* = 0.308) indicated no significant publication bias (Supporting Figure 1).

### 3.4. HMGB1 in Serum

**FIGURE 3:**
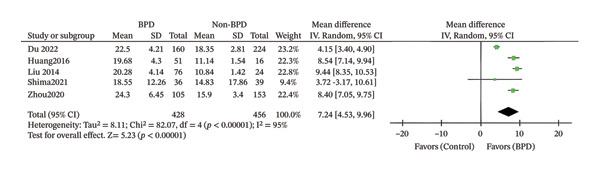
Forest plot comparing serum HMGB1 levels between the BPD and non‐BPD groups using a random‐effects model.

Five studies evaluated HMGB1 levels in serum. A high degree of heterogeneity was observed (*I* ^2^ = 95%, *p* < 0.0001). Using a random‐effects model, the pooled MD was 7.24 ng/mL (95% CI = 4.53–9.96; *Z* = 5.23; *p* < 0.00001), indicating significantly higher HMGB1 levels in the BPD group than in the control group (Figure 3).

Sensitivity analysis (Supporting Table 2) identified the study by Du Weiwei et al. [24] as a principal source of heterogeneity. After excluding this study, *I* ^2^ decreased from 95% to 23% (*p* = 0.28), and the pooled effect size was recalculated (MD = 8.84; 95% CI = 8.12–9.56; *p* < 0.00001) under a fixed‐effects model (Supporting Figure 2). Subsequent subgroup analyses after excluding this study produced results consistent with the overall findings, further supporting the robustness of the serum HMGB1 association.

Publication bias was assessed using funnel plot symmetry and Begg’s test. The funnel plot appeared symmetric, and Begg’s test yielded *p* > 0.99, indicating no significant publication bias (Supporting Figure 3).

### 3.5. Subgroup Analysis

**FIGURE 4:**
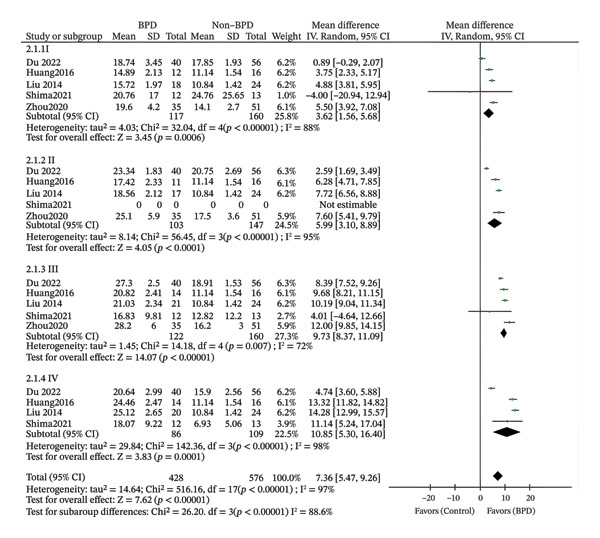
Forest plot comparing serum HMGB1 levels between the BPD and non‐BPD groups across four stages.

To explore potential sources of heterogeneity, subgroup analyses were performed according to postnatal stage. Serum HMGB1 concentrations were significantly higher in neonates with BPD than in those without BPD across the four stages (Stages I–IV). Stage I, MD = 3.62 ng/mL (95% CI, 1.56–5.68; *p* = 0.0006); Stage II, MD = 5.99 ng/mL (95% CI, 3.10–8.89; *p* < 0.00001); Stage III, MD = 9.73 ng/mL (95% CI, 8.37–11.09; *p* < 0.00001); and Stage IV, MD = 10.85 ng/mL (95% CI, 5.30–16.40; *p* = 0.0001). Substantial heterogeneity persisted within all stage‐specific analyses (*I* ^2^ = 72%–98%; Figure 4 and Table 2, panel A).

**TABLE 2:** Aggregate serum HMGB1 concentrations, stage‐specific meta‐analyses, and pairwise temporal comparisons across four postnatal stages.

Panel A. Between‐group meta‐analysis by postnatal stage							
Stage	No. of studies	BPD, *n*	BPD‐weighted mean ± pooled SD (ng/mL)	Non‐BPD, *n*	Non‐BPD‐weighted mean ± pooled SD (ng/mL)	Pooled MD (95% CI), ng/mL	*p* value; I2 (%)
I	5	117	18.34 ± 6.24	160	15.49 ± 7.43	3.62 (1.56–5.68)	0.0006; 88
II	4	109	22.52 ± 3.81	147	16.96 ± 2.81	5.99 (3.10–8.89)	< 0.00001; 95
III	5	128	24.71 ± 4.81	160	15.56 ± 3.97	9.73 (8.37–11.09)	< 0.00001; 72
IV	4	92	21.95 ± 4.27	109	13.02 ± 2.67	10.85 (5.30–16.40)	0.0001; 98

**Panel B. Pairwise temporal comparisons within each group**							

**Comparison**		**BPD MD (95% CI), ng/mL**		**p** **(BPD)**	**Non-BPD MD (95% CI), ng/mL**		**p** **(non-BPD)**

I vs. II		−3.77 (−5.04 to −2.50)		< 0.00001	−1.56 (−3.33–0.21)		0.08
I vs. III		−6.74 (−8.66 to −4.82)		< 0.00001	−0.73 (−1.65–0.20)		0.13
I vs. IV		−5.79 (−10.66 to −0.92)		0.02	0.85 (−0.67–2.38)		0.27
II vs. III		−3.45 (−4.16 to −2.75)		< 0.00001	0.78 (−0.22–1.78)		0.13
II vs. IV		−3.61 (−10.52 to 3.31)		0.31	1.61 (−1.52–4.75)		0.31
III vs. IV		−0.52 (−7.16 to 6.13)		0.88	1.33 (−0.69–3.35)		0.20

*Note:* Values in Panel A are descriptive sample‐size‐weighted means ± pooled within‐study SDs. Weighted means were calculated using study‐specific sample sizes, and pooled SDs were derived from study‐specific within‐group variances. Stage‐specific MDs and 95% CIs were estimated using random‐effects meta‐analyses. Because the descriptive weighted means and meta‐analytic MDs were calculated using different weighting methods, the arithmetic difference between the weighted means may not equal the pooled MD. Stage‐specific sample sizes represent observations rather than unique infants, because some studies contributed repeated measurements from the same infants across multiple stages. In Panel B, MD was calculated as the concentration at the earlier stage minus that at the later stage; therefore, negative values indicate higher HMGB1 concentrations at the later stage. Temporal comparisons should be interpreted cautiously because some studies included repeated measurements from the same infants across stages. Stage I, postnatal days 1–3; Stage II, days 4–10; Stage III, days 10–20; Stage IV, > 20 days. BPD, bronchopulmonary dysplasia.

Abbreviations: CI, confidence interval; HMGB1, high‐mobility group box 1; MD, mean difference; SD, standard deviation.

The descriptive sample‐size‐weighted mean serum HMGB1 concentration in the BPD group increased from 18.34 ng/mL at Stage I to 22.52 ng/mL at Stage II and 24.71 ng/mL at Stage III, whereas the corresponding value at Stage IV was 21.95 ng/mL. No consistent increasing pattern was observed in the non‐BPD group (Table 2, panel A).

**FIGURE 5:**
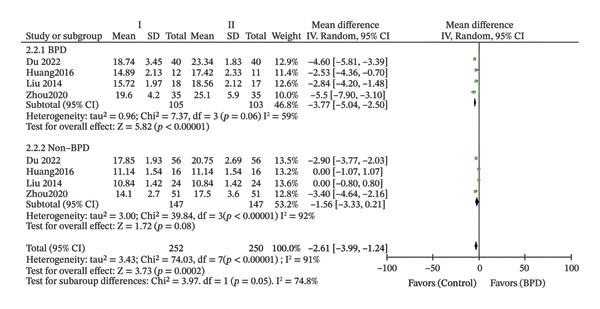
Forest plot comparing serum HMGB1 levels between the BPD and non‐BPD groups in stages I and II.

Pairwise temporal comparisons showed that, within the BPD group, serum HMGB1 concentrations were significantly higher at Stage II than at Stage I (MD for Stage I minus Stage II, −3.77 ng/mL; 95% CI, −5.04 to −2.50; *p* < 0.00001), at Stage III than at stage I (MD, −6.74 ng/mL; 95% CI, −8.66 to −4.82; *p* < 0.00001), and at Stage III than at Stage II (MD, −3.45 ng/mL; 95% CI, −4.16 to −2.75; *p* < 0.00001). Stage IV concentrations remained significantly higher than those at Stage I but did not differ significantly from those at stages II or III. No significant pairwise temporal differences were observed in the non‐BPD group (all *p* ≥ 0.08; Table 2, panel B; Figure 5).

Collectively, these exploratory temporal analyses suggest that serum HMGB1 concentrations increased during the first 20 postnatal days in neonates with BPD, with no statistically detectable further increase after Stage III. These findings should be interpreted cautiously because some studies contributed repeated measurements from the same infants, and the available data did not permit consistent adjustment for within‐infant correlations.

## 4. Discussion

This systematic review and meta‐analysis showed that total HMGB1 concentrations in serum and BALF were higher in neonates with BPD than in those without BPD. However, this association does not establish disease specificity or demonstrate that HMGB1 can distinguish BPD from other neonatal pulmonary or systemic inflammatory conditions. Moreover, because the included studies measured total HMGB1 without differentiating among redox isoforms, the observed concentration differences cannot be interpreted as direct evidence of increased pro‐inflammatory HMGB1 activity. Furthermore, the temporal fluctuations in HMGB1 levels across different stages of BPD suggest its potential as a marker of therapeutic efficacy. Notably, the elevation of HMGB1 concentrations observed throughout postnatal stages of BPD highlights its promise as an early biomarker for disease detection and prognosis. However, no significant elevation was observed in Stage IV (> 20 days). These results underscore the need for further investigation into the mechanistic pathways through which HMGB1 contributes to lung injury and the development of targeted therapies aimed at modulating its activity in neonates at risk for BPD.

This study demonstrated that HMGB1 levels in both serum and BALF were significantly higher in the BPD group than in the control group, implicating HMGB1 in the pathogenesis of BPD. As a prototypical damage‐associated molecular pattern protein, HMGB1 is actively released from alveolar epithelial cells in response to injury or oxidative stress. After reaching the extracellular environment, HMGB1 signals through a broader receptor repertoire, including TLR2, TLR4, TLR9, and RAGE, often in cooperation with nucleic acids or other ligands, culminating in canonical and the noncanonical activation of NF‐κB and transcription of IL‐1β, TNF‐α, and IL‐6, among others [25, 26]. This multiple‐receptor network helps explain the persistent, sterile inflammation observed in BPD. Previous studies found that HMGB1 blockade can mitigate the pathology of BPD. For instance, Lu et al. [27] reported that HMGB1‐targeting peptides attenuate collagen deposition in BALF and ameliorate alveolar simplification by suppressing hyperoxia‐induced inflammatory mediators, such as IL‐13. Moreover, single‐cell transcriptomic analyses revealed that HMGB1 modulates the aberrant proliferation and polarization of alveolar macrophages, partly through activation of the IL‐33–ST2 axis, a pathway closely associated with impaired alveolar development [28]. These findings align with our results and reinforce the hypothesis that HMGB1 contributes to BPD progression by exacerbating inflammatory responses and disrupting alveolar repair mechanisms.

The biological activity of extracellular HMGB1 is strongly influenced by its redox state. All‐thiol HMGB1 primarily promotes chemotaxis, disulfide HMGB1 induces cytokine release, and terminally oxidized HMGB1 is generally considered biologically inactive. The immunoassays used in the included studies quantified total HMGB1 and did not distinguish among these functionally distinct isoforms. Therefore, the pooled elevation of total HMGB1 does not identify the predominant redox form in BPD or establish that the net biological activity of HMGB1 is pro‐inflammatory. Studies using redox‐sensitive assays are needed to characterize HMGB1 isoforms in neonatal serum and BALF and to determine their associations with the development and severity of BPD.

Despite this analytical limitation, preclinical lung injury models relevant to BPD have shown that pharmacologic HMGB1 inhibition can reduce inflammation and improve lung structure [29, 30]. Notably, glycyrrhizin, a natural triterpenoid that directly binds HMGB1 and prevents receptor engagement, mitigates cytokine production and alveolar injury [31]. Gabexate mesilate, a serine protease inhibitor that suppresses HMGB1 release and downstream signaling, similarly attenuates lung inflammation and edema [32, 33]. Together with evidence that HMGB1 blockade (*e.g.*, BoxA/anti‐HMGB1) dampens TLR/RAGE NF‐κB signaling [33, 34] and collagen deposition [35], these data support HMGB1 as a credible therapeutic target warranting clinical translation in neonates at risk of BPD.

Subgroup analysis revealed an association between dynamic changes in HMGB1 levels and the progression of BPD. Although significant increases in HMGB1 levels were observed in the BPD group up to 20 days, no significant fluctuations were noted in Stage IV (> 20 days). By contrast, no significant changes in HMGB1 levels were observed in the control group across the postnatal stages. This progressive elevation might represent a biomarker reflecting both the stage and severity of the disease. In support of this, Hara et al. [16] reported that BALF HMGB1 concentrations increased markedly within 72 h after hyperoxia exposure in a BPD animal model and remained elevated beyond 14 days. Similarly, Ulinder et al. [36] found that serum HMGB1 levels in preterm infants increased before the clinical diagnosis of BPD, and BPD levels were correlated with deterioration of the alveolar‐arterial oxygen gradient. These dynamic changes in HMGB1 expression might reflect its biphasic role in BPD pathogenesis: Early release amplifies the inflammatory cascade, whereas prolonged elevation impairs alveolar epithelial cell differentiation, particularly the transdifferentiation of alveolar type II cells to type I cells [37], ultimately contributing to arrested alveolarization.

Given the increased levels of HMGB1 in BPD, it represents a promising therapeutic target. Several strategies have been proposed to modulate its pathological activity. The first strategy is HMGB1 antagonism. Preclinical studies demonstrated that HMGB1‐neutralizing antibodies or peptides (such as BoxA) attenuate inflammation and improve alveolar architecture by disrupting HMGB1–TLR4/RAGE signaling [38]. The administration of HMGB1 peptides to hyperoxia‐exposed mice reduced IL‐13 expression and collagen deposition in BALF, thereby ameliorating lung injury [27]. The second strategy is epigenetic regulation. Post‐translational modifications, including acetylation and phosphorylation, are critical for HMGB1 translocation and extracellular release, whereas the cysteine redox status dictates receptor‐specific activity (all‐thiol: chemotactic; disulfide: cytokine‐inducing; terminally oxidized: inactive). Therapeutic approaches that limit nuclear export (*e.g.*, deacetylase activation), prevent disulfide formation, or neutralize extracellular HMGB1 might therefore differentially modulate inflammation in BPD [16]. Modulation of these processes through deacetylase activators (e.g., SIRT1) or kinase inhibitors can suppress HMGB1‐mediated inflammation and mitigate lung damage [39]. The third strategy is combination therapy. In models of BPD complicated by pulmonary hypertension, co‐administration of HMGB1‐targeting agents with 5′‐adenosine monophosphate‐activated protein kinase activators, such as 5‐aminoimidazole‐4‐carboxamide ribonucleoside, synergistically reduced alveolar macrophage‐driven inflammation and enhanced autophagy‐mediated repair mechanisms [40]. Collectively, these findings support the potential of HMGB1‐directed interventions as a novel therapeutic avenue for BPD, particularly when integrated into multimodal treatment strategies.

HMGB1 might also serve as a diagnostic biomarker for BPD. In this study, HMGB1 levels in both serum and BALF tended to be elevated in infants with BPD, indicating its potential utility in auxiliary diagnosis. Moreover, combining BALF HMGB1 detection with other inflammatory mediators (such as IL‐4 and IL‐13) could improve the early identification of alveolar injury and inflammation [41]. In hyperoxia‐induced animal models of BPD, HMGB1 levels were inversely correlated with both BALF collagen content and static lung compliance, further underscoring the association of HMGB1 with structural and functional lung impairment [42]. However, no clinically validated diagnostic threshold for HMGB1 has been established. Large, multicenter cohort studies are therefore required to define clinically relevant cut‐off values and evaluate its diagnostic performance alongside established clinical indicators, such as the duration of oxygen dependence and measures of alveolar simplification.

Importantly, HMGB1 is released in response to tissue injury and innate immune activation and is not specific to BPD. Elevated concentrations may also occur in neonatal pneumonia, sepsis, acute lung injury, and other inflammatory conditions. The available evidence therefore does not establish that total HMGB1 can independently distinguish BPD from other causes of pulmonary or systemic inflammation. In addition, because existing assays do not differentiate among HMGB1 redox isoforms, which have distinct biological activities, total HMGB1 concentrations alone cannot reliably indicate its net inflammatory activity. Future diagnostic studies should include appropriate disease‐control groups, incorporate isoform‐sensitive assays, and determine whether HMGB1 provides incremental diagnostic or prognostic value beyond established clinical predictors and other biomarkers.

Several limitations of this study warrant consideration. First, substantial heterogeneity was observed across the included studies, potentially arising from differences in sample type, participant characteristics, and BPD severity or classification. Second, most studies compared infants with BPD with non‐BPD controls but did not include disease‐control groups with neonatal pneumonia, sepsis, or other inflammatory pulmonary conditions. Consequently, this meta‐analysis could not assess the disease specificity of HMGB1 or its ability to differentiate BPD from other causes of pulmonary or systemic inflammation. Third, the assays used in the included studies measured total HMGB1 without distinguishing among the all‐thiol, disulfide, and terminally oxidized isoforms. As these redox isoforms exert distinct biological effects, total HMGB1 concentrations cannot define the net inflammatory activity of HMGB1 or identify the isoform most closely associated with BPD. Fourth, insufficiently detailed data on gestational age and the diagnostic definitions of BPD precluded subgroup analyses according to these factors. Finally, although the sensitivity analyses indicated that the overall findings were relatively stable, publication bias cannot be excluded and may have resulted in an overestimation of the association between HMGB1 and BPD. Future research should prioritize multicenter, prospective studies to further investigate the diagnostic and therapeutic potential of HMGB1 in the clinical management of BPD. Clinical trials evaluating HMGB1‐targeting interventions must address safety profiles and long‐term outcomes in preterm infants. Additionally, integrating single‐cell transcriptomics and proteomics could provide deeper insight into the dynamic regulatory networks involving HMGB1, particularly its role in alveolar epithelial–mesenchymal crosstalk and lung development.

## 5. Conclusions

This systematic review and meta‐analysis indicates that total HMGB1 concentrations are elevated in neonates with BPD and may reflect disease‐associated inflammatory lung injury. However, HMGB1 is not specific to BPD, and the assays used in the included studies did not distinguish among redox isoforms with distinct biological activities. These limitations restrict the use of total HMGB1 as a standalone diagnostic biomarker and preclude direct inference regarding its pro‐inflammatory activity. Future prospective studies should include appropriate disease‐control groups, use redox‐sensitive assays, and determine whether HMGB1 provides incremental diagnostic or prognostic value beyond established predictors of BPD. Further research is also needed to define clinically relevant thresholds, elucidate the molecular pathways linking specific HMGB1 isoforms to impaired lung development, and evaluate the safety and efficacy of HMGB1‐targeted interventions. Collectively, the available evidence supports HMGB1 as a candidate biomarker and potential therapeutic target in BPD, although its clinical utility remains to be established.

**Nomenclature**
High‐mobility group box 1: HMGB1
Bronchopulmonary dysplasia: BPD
Bronchoalveolar lavage fluid: BALF
Mean difference: MD
Confidence interval: CI
Standard deviation: SD

## Author Contributions

Hejia Li: roles/writing–original draft.

Jiao Li: formal analysis.

Shanshan Li: data curation.

Xiaojuan Huang: data curation.

Shuaiyi Li: data curation.

Wenhui Wu: supervision.

Yuqing Fan: supervision.

Yurun Wang: software.

Yue Zhang: validation.

Jiashun Liu: software.

Lu Zou: validation.

ChangJun Ren: writing–review and editing.

Shangbin Li: project administration.

## Funding

This study was supported by the Medical Science Research Program of the Health Commission of Hebei Province, China (Grant No. 20250027).

## Conflicts of Interest

The authors declare no conflicts of interest.

## Supporting Information

Additional supporting information can be found online in the Supporting Information section.

## Supporting information


**Supporting Information** Supporting Table 1. Sensitivity analysis of studies measuring HMGB1 levels in BALF among neonates with BPD. Supporting Table 2. Sensitivity analysis of studies measuring serum HMGB1 levels among neonates with BPD. Supporting Figure 1. Funnel plot of the included studies assessing HMGB1 levels in BALF to evaluate heterogeneity. Supporting Figure 2. Forest plot comparing serum HMGB1 levels between the BPD and non‐BPD groups using a fixed‐effects model (excluding Du Weiwei et al.). Supporting Figure 3. Funnel plot of the included studies assessing HMGB1 levels in serum to evaluate heterogeneity.

## Data Availability

The authors confirm that the data supporting the findings of this study are available within the article.
