# Supplementary material for: Elevated HMGB1 Levels in Neonates With Bronchopulmonary Dysplasia: A Systematic Review and Meta‐Analysis
Source: Can Respir J. 2026 Aug 13;2026:2816142. doi: 10.1155/carj/2816142 (PMC13470612; doi:10.1155/carj/2816142)
Supplement: Supplementary file 1 [file CARJ-2026-2816142-s001.docx]

| Supplementary Table 1. Sensitivity analysis of studies measuring HMGB1 levels in BALF among neonates with BPD | | | | | | |
| --- | --- | --- | --- | --- | --- | --- |
| Excluded study (Author, year) | *I^2^* (%) | P | Mean difference | 95% CI | Z | P |
| Aghai, 2010 | 83% | 0.003 | 4.57 | (3.86–5.27) | 12.72 | <0.00001 |
| Cheng, 2016 | 82% | 0.004 | 5.53 | (4.40–6.65) | 9.65 | <0.00001 |
| Huang, 2016 | 91% | <0.0001 | 5.23 | (3.88–6.58) | 7.60 | <0.00001 |
| Liu, 2014 | 91% | <0.0001 | 5.36 | (3.86–6.86) | 7.00 | <0.00001 |

CI, confidence interval

| Supplementary Table 2. Sensitivity analysis of studies measuring serum HMGB1 levels among neonates with BPD. | | | | | | |
| --- | --- | --- | --- | --- | --- | --- |
| Excluded study (Author, year) | I^2^ (%) | P | Mean difference | 95%CI | Z | P |
| Du, 2022 | 23% | 0.28 | 8.84 | (8.12–9.56) | 24.03 | <0.00001 |
| Huang, 2016 | 96% | <0.00001 | 6.84 | (3.50–10.17) | 4.01 | <0.0001 |
| Liu, 2014 | 94% | <0.00001 | 6.61 | (3.62–9.59) | 4.34 | <0.0001 |
| Shima, 2021 | 96% | <0.00001 | 7.61 | (4.73–10.48) | 5.19 | <0.00001 |
| Zhou, 2020 | 96% | <0.00001 | 6.87 | (3.48–10.25) | 3.97 | <0.0001 |

Supplementary Figure 1. Funnel plot of the included studies assessing HMGB1 levels in BALF to evaluate heterogeneity.

Supplementary Figure 2. Forest plot comparing serum HMGB1 levels between the BPD and non-BPD groups using a fixed-effects model (excluding Du Weiwei *et al.*).

Supplementary Figure 3. Funnel plot of the included studies assessing HMGB1 levels in serum to evaluate heterogeneity.
